# Shame for disrespecting evidence: the personal consequences of insufficient respect for structural equation model testing

**DOI:** 10.1186/1471-2288-14-124

**Published:** 2014-11-27

**Authors:** Leslie A Hayduk

**Affiliations:** Department of Sociology, University of Alberta, Edmonton, Canada

**Keywords:** Factor analysis, Factor model, Testing, Close fit, Structural equation model, SEM

## Abstract

**Background:**

Inappropriate and unacceptable disregard for structural equation model (SEM) testing can be traced back to: factor-analytic inattention to model testing, misapplication of the Wilkinson task force’s [*Am Psychol* 54:594-604, 1999] critique of tests, exaggeration of test biases, and uncomfortably-numerous model failures.

**Discussion:**

The arguments for disregarding structural equation model testing are reviewed and found to be misguided or flawed. The fundamental test-supporting observations are: a) that the null hypothesis of the χ^2^ structural equation model test is not nil, but notable because it contains substantive theory claims and consequences; and b) that the amount of covariance ill fit cannot be trusted to report the seriousness of model misspecifications. All covariance-based fit indices risk failing to expose model problems because the extent of model misspecification does not reliably correspond to the magnitude of covariance ill fit – seriously causally misspecified models can fit, or almost fit.

**Summary:**

The only reasonable research response to evidence of non-chance structural equation model failure is to diagnostically investigate the reasons for failure. Unfortunately, many SEM-based theories and measurement scales will require reassessment if we are to clear the backlogged consequences of previous deficient model testing. Fortunately, it will be easier for researchers to respect evidence pointing toward required reassessments, than to suffer manuscript rejection and shame for disrespecting evidence potentially signaling serious model misspecifications.

## Background

Rodgers [1] compiled a history of null hypothesis significance testing and reported a “quiet revolution” that “obviated” many of the criticisms previously leveled against testing. Rodger’s article appeared in the *American Psychologist* – the same journal that a decade earlier carried a frequently cited critique of testing by Wilkinson and the Task Force on Statistical Inference [2]. Rodger’s modeling discussion was sufficiently muted that readers could have easily slept through the revolution. The current article extends the testing discussion as it applies *to structural equation models* (SEM). SEMNET [3] distributed heated discussions of structural equation model testing to a somewhat limited audience, but the model-testing revolution was sufficiently swift that recapitulation will likely assist anyone who blinked.

The χ^2^ test of structural equation models became widely available with the appearance of LISREL-III in 1976 [4]. Some researchers began employing structural equation model testing shortly thereafter [5]. Other researchers – often those using factor-structured models – tended not to test their models. It took some time for other factor analytic programs to provide the test, but the primary impetus for disregard of model testing was researchers’ dislike of reporting their model’s failure [6]. The laxity of factor model testing was one of many analytical differences between factor modeling and more general structural equation modeling but the factor-SEM differences remained non-contentious as long as each modeling style played in its own academic sandbox. Things changed with the arrival of SEMNET [3], a free internet listserv hosted at the University of Alabama and devoted to structural equation modeling, including factor modeling. SEMNET discussions of the frictions between factor and SEM procedures were sufficiently heated that they led to a special issue of *Structural Equation Modeling* in 2000. The special issue’s target article by Hayduk and Glaser [7] devoted several pages to model testing, which prompted additional SEMNET debate – more accurately dissention – which in turn spawned a special issue of *Personality and Individual Differences* devoted to SEM testing via Barrett’s [8] target article. The only alternative to model testing is model fit indexing, and Barrett’s overall assessment of indexing was not particularly gentle: “In fact, I would now recommend banning *ALL* such indices from ever appearing in any paper as indicative of model “acceptability” or “degree of misfit”.” [8]:821. Barrett ended his article saying: “But, if SEM is used, then model fit testing and assessment is paramount, indeed crucial, and cannot be fudged for the sake of “convenience” or simple intellectual laziness on the part of the investigator.” [8]:823. Barrett’s article also included milder, mollifying, statements but these were dismantled in the commentary by Hayduk, Cummings, Boadu, Pazderka-Robinson, and Boulianne [9].

With this historical backdrop, we can consider and reassess the arguments employed to circumnavigate structural equation model testing.

## Discussion

### Argument 1: the nil null

In many testing contexts the null hypothesis is a nil hypothesis because it corresponds to no effect, no relationship, or no correlation. This makes the null hypothesis nil in the sense that it focuses on a lack or absence of something, and subsequently fails to correspond to a feature of theoretical interest.

The null hypothesis in structural equation model testing is not nil because it encapsulates a theory’s claims about the structures producing the observed variables. The SEM null hypothesis is as substantive and notable as is the modeled theory. The notable-null hypothesis of the χ^2^ model test is the covariance matrix implied by the model when all the theory’s constraints have been respected, and the theory has been enhanced by optimal estimates of all the theorized effects [10]:256, [11]:160. Each theorized/modeled effect is given an optimal estimate, each theorized absence-of-effect is respected, and all the indirect consequences of these theorized features contribute to calculating the model-implied covariance matrix – which is usually designated as Σ, or Σ(θ) where θ is the vector of coefficient estimates. The χ^2^ test examines whether the observed data covariance matrix differs significantly (namely by more than what might reasonably be attributed to chance variations) from the specific covariance matrix demanded by the estimate-enhanced theory in the model. Thus, structural equation model testing employs a notable-null hypothesis, not a nil-null hypothesis.

Individual coefficients within structural equation models can be tested, but whether such tests involve nil-nulls or notable-nulls depends on the theory’s requirements and how the testing is done. Though relatively rarely employed, effect or other coefficients assigned fixed non-zero values (which constitute notable-nulls) can be tested using χ^2^ difference tests [10–12]. Estimated effect coefficients, on the other hand, typically employ nil-nulls in the sense that zero constitutes the tested null hypothesis. But even zero-nulls for estimated SEM coefficients may not constitute nil-nulls because an effect estimate may be investigating whether an effect is zero after having controlled for a multitude of theorized model features (e.g. indirect effects, common causes, or feedback). Investigating whether multiple theory-based controls render some specific effect zero, or “unnecessary,” constitutes an investigation of theoretical-structuring, which renders even a zero-effect hypothesis substantive and non-nil. From the perspective of model testing, the important observation is that the χ^2^ test investigates whether the data is consistent with the model’s/theory’s constraints and claims as incorporated in the Σ matrix – which renders the tested hypothesis as notable as is the theory itself.

## Background to the next arguments

Several of the excuses for avoiding structural equation model testing evaporate once it is understood that *the amount of ill fit between the data covariance matrix and the model-implied covariance matrix Σ does NOT assuredly, consistently, or reliably correspond to the seriousness of the problems in a model’s specification*.

There are several ways to illustrate this. Improper causal directions are undeniably causal misspecifications but notice that reversing the direction of some modeled causal effects may, *or may not*, alter a models’ ability to fit the data. Models fitting covariance data equally well are traditionally referred to as equivalent models, though they are more accurately described as “fit-equivalent but causally-different” models. If a specific causal direction is correct and provides perfect-fit, an equivalent model containing the opposite causal direction would constitute a causally misspecified model displaying equivalently-perfect fit.

For a second way to see why seriously causally wrong models can fit, notice that saturated models (namely, models having as many estimated coefficients as modeled covariances) often fit perfectly – without requiring the model to be properly causally structured. Replacing near-zero estimated coefficients, with fixed zeros, in such models produces non-saturated models that fit – with no requirement that the model is even close to properly causally specified.

Exploratory factor models provide a third way to understand how causally misspecified models can fit. Increasing the number of factors progressively improves fit, and somewhere at or below the maximum number of estimable factors (the Ledermann bound [13]) the factor model, possibly supplemented with error covariances, is likely to provide nearly perfect covariance fit – whether or not the causal world that generated the factored items was factor structured. Since non-factor causal worlds can differ importantly from factor-structured models, the progressive fit improvement provided by additional factors illustrates how models might achieve fit despite being seriously causally misspecified.

If you are not persuaded by these statistical observations, you might consider the examples of factor models that provide perfect covariance fit despite being seriously causally misspecified, even when the factor models are not-saturated, not-equivalent, and not at the Ledermann-bound [14], as well as the simulations by Savalei [15] and Heene, Hilbert, Freudenthaler and Buhner [16].

If a seriously-causally-wrong model provides perfect covariance fit, slight changes to that model will result in only minor amounts of covariance ill fit. Consequently even a small amount of covariance ill fit may signal serious model misspecification. Researchers should therefore be alert to the possibility of important model causal misspecification irrespective of how much, or how little, ill covariance fit a model displays. Small random amounts of ill covariance fit might arise from random sampling variations but any additional (beyond random) ill covariance fit may, or may not, be signs of serious model misspecification. Once the amount of covariance ill fit exceeds what might reasonably result from random variations (which is what χ^2^ tests), the appropriate research response is to thoroughly investigate the possible sources of the ill fit – including the possibility of major model misspecifications. The researcher’s task is to evaluate the model, not merely to evaluate the fit provided by that model – as is exemplified by rejection of fitting models if they contain wrong-signed estimates. With this in mind, we return to considering the arguments regarding SEM testing.

### Argument 2: use confidence intervals instead of tests

Most structural equation modeling programs report several indices of the fit between the model-implied and data covariance matrices, and a confidence interval can be calculated for the RMSEA index [17]. The fundamental problem confronting the RMSEA and all fit indices is that we cannot trust that the indexed amount of ill fit displayed by a model corresponds to the seriousness of the model’s specification problems. Even extremely close covariance fit – whether the close-fit is assessed by an index value, or confidence interval around an index value – may have resulted from a model containing multiple serious misspecifications.

The fact that the feature of concern to the researcher (serious model misspecification) cannot be trusted to correspond to the indexable magnitude of covariance ill fit, devastates the model assessment capabilities of all the covariance-based fit indices, with or without their confidence intervals. Once the amount of ill fit exceeds what might reasonably be attributable to chance sampling variations, the researcher must acknowledge and diagnostically investigate the possibility of serious model misspecifications – no matter the amount or magnitude of the ill covariance fit. The simple fact that the feature of interest to the researcher (model causal misspecification) does not confidently correspond to any index’s magnitude renders all the fit indices untrustworthy, whether or not the index has a confidence interval. Confidence intervals are useful with features like correlations because the feature of interest to the researcher and the estimated correlation’s magnitude correspond. It is the gap between the fit index values and the structural equation modeling feature of interest (model misspecification) that renders fit indices untrustworthy reporters of model structural adequacy.

Anyone looking closely at index-supporting articles, such as Browne and Cudeck’s [17] discussion of the RMSEA, will find that the *supposedly*-acceptable range of index values requires that the researcher overlook some amount of *real* (not likely to be chance) ill fit. For example, when Browne and Cudeck [17] suggest it is OK to overlook some real (non-chance) amount of “error of approximation” they are inviting (actually deceptively enticing) researchers to disregard real evidence which may be signaling serious and important model misspecifications. Supplementing the RMSEA with another index, or moving to pairs of indices, can detect some very simple coefficient misspecifications in factor models [18], but there seems little prospect that even a team of covariance based indices, with or without confidence intervals, will ever confidently detect more complex causal misspecifications, such as models containing wrong latents as well as wrong coefficient interconnections. When a factor model provides perfect covariance-fit to data originating from a non-factor causal world [14], *all* the covariance based indices will report “fit” and hence even all the indices together would be unable to detect the model’s causal misspecification. If a researcher is so lax as to “permit” confidence intervals around the indices, some additional amount of real ill covariance fit would also be “permitted” and hence additional misspecified models would go undetected by assessments appealing to “supposedly-acceptable” values on pairs, triplicates, or even lists of fit indices.

### Argument 3: too much power for practicality

When examining effects or correlations the standard error of the estimate decreases as *N* increases. Consequently, as sample size increases, progressively smaller correlations or effect estimates become statistically significant – namely will be evidenced as unlikely to have arisen by mere chance sampling variations. This leads to the objection that as *N* increases, trivially small and useless correlations or effects can be found to be statistically significant.

Switching to structural equation modeling, we again remember that it is possible for seriously causally misspecified models to display no ill fit at all, and hence that seriously misspecified models may display: no ill fit, a small amount of ill fit, or a huge amount of covariance ill fit. It is true that increasing *N* makes it easier to detect smaller amounts of covariance ill fit as unlikely to be chance sampling artifacts, but it constitutes a statistical mistake to claim that a small amount of observed covariance ill fit says the model contains only small or trivial misspecifications. The fact that important model misspecifications may result in absolutely no ill fit at all [14], renders the small-is-trivial or small-is-useless objection statistically inappropriate in the context of testing structural equation models. It is not uncommon for researchers to brandish a style of misspecification that would not concern them (e.g small cross-loadings) as their “justification” for failing to attend to significant model ill fit. Unfortunately, such claims are empty distractions unless the researcher’s diagnostic investigations point to the selected reason, as opposed to some more devastating reason, as constituting the actual source of the ill fit.

Increasing *N* increases the power to detect many, but not all, kinds of model misspecifications. In other words, increasing *N* reduces the probability of making a Type II error, which in this case would be the error of accepting a model when in fact that model is causally misspecified. The first step to correcting model misspecification is to detect the misspecification, and increasing *N* increase the power to detect some kinds of model misspecifications.

### Argument 4: all models will fail if N is large enough

Argument 3 is often expressed slightly differently in the context of structural equation models – namely as Argument 4, the (erroneous) claim that all structural equation models will fail the χ^2^ test if *N* is large enough. In this context, Box is often cited as saying “all models are wrong but some are useful” [19]:202, [20]:424, see also [21]:2. There are two rebuttals to this claim, one philosophical, the other statistical.

The philosophical rebuttal questions how anyone might confidently know that all models are wrong, and therefore that none will ever be correct or proper. No one has yet seen, and no one is ever likely to see, all the possible models that can be specified in all possible research contexts. Hence, the claim is based on the speaker’s assessment of the ultimate capabilities of scientific endeavors, and such an assessment invokes philosophical speculations quite removed from statistical or practical research considerations. Practicality simply asks: Does the current data provide evidence of model problems? The practical answer being: Sometimes it does, and sometimes it doesn’t.

The statistical response to the claim that all structural equation models will fail if *N* is large enough, points to a statistical mistake easily connected to the formula for the model χ^2^ test. The formula for the maximum likelihood χ^2^ is the product of two parts.


where the first part directly depends upon sample size.


Here *N* is the sample size minus one [22]. The *N* on the right side of the equation makes it easy to presume that χ^2^ will unavoidably increase as *N* increases. Actually, χ^2^ may or may not increase with increasing *N*. There will be no increase in χ^2^ for properly specified (non-problematic) models, because Part-2 of the equation will take on a value that on average equals the degrees of freedom of the model divided by *N*.


Thus, no matter how big *N* gets, the *N* from the numerator cancels the *N* in the denominator, and χ^2^ maintains a value within sampling variations of the model degrees of freedom, exactly as it should. Many simulations do not report χ^2^ for the true model but whenever they do, the maximum likelihood χ^2^ behaves as it should and does not increase with *N* (e.g. [23]: Table three).

For some problematic or misspecified models, the average of Part-2 of the formula is not exactly *df/N* as above, and it does not decline as quickly as *N* increases. Consequently, χ^2^ tends to increase with *N* and thereby displays increasing power to detect these problematic models. Thus, when a model’s χ^2^ increases with *N*, that signals something is detectably-problematic about the model.

A fundamental assumption of structural equation models is that the sampled cases are causally homogeneous and responsive to the same causal forces. That assumption becomes progressively more tenuous as *N* increases because it becomes increasingly likely that some cases are embedded in different causal contexts than are other cases. Consequently, as *N* increases, models are prone to becoming causally misspecified because they do not accurately represent all the modeled cases. This style of model failure is not *N*’s fault. Such a failure is the consequence of the researcher having inappropriately applied their model to causally non-homogeneous cases. Increasing *N* may also improve detection of nonlinearities, failure to attain interval level measurement, non-normality, and error non-independence, along with a multitude of other kinds of model misspecifications; so significant ill fit should be supplemented with diagnostic investigations aimed at clarifying which problem or problems have been encountered. Statistically significant ill fit indicates non-chance diagnostic evidence is available, and this evidence should be investigated carefully. For example, it would be unreasonable to point to non-normality as the culprit unless the ill fit was attached to the least-normal variables, or that ordinal measurement was the culprit unless the ill fit coordinated with the most problematic measures. For problematic models, increasing *N* not only increases the likelihood of detecting model problems, it increases the possibility of diagnostically locating and redressing the problems because the trail leading from specific ill fit covariances back toward the problematic-source becomes more stable and diagnostically dependable.

Authors inclined to cite Box as saying “all models are wrong” should notice that Box differentiated between models as curve fitting (or fitting a response surface), from the more difficult task of modeling “how a system works” [24]:57. For Box it was a more scientifically informative, even if “hazardous undertaking”, to find out “what the world is really like” [21]:1. Box was very clear when he said: “Since all models are wrong the scientist must be alert to what is importantly wrong. It is inappropriate to be concerned about mice when there are tigers abroad” [25]:792. In SEM, tigers = model causal misspecification = importantly wrong. A scientist using structural equation modeling should indeed “be alert to what is importantly wrong”, lest they succumb to a Boxian tiger.

### Argument 5: fishing

If a researcher uses the traditional 0.05 *p* value and tests multiple effects or correlations, the researcher should expect about one out of every 20 tests to be significant at the 0.05 level by chance alone when all the effects or correlations are truly zero. Selectively reporting significant effects obtained via multiple testings introduces testing-bias that can be adjusted for via Bonferroni and other methods [26]. This concern applies to structural equation modeling in three different ways, one of which is moderately problematic.

First, a concern for fishing might apply to the multiple coefficients estimated within a model. This problem is not particularly acute because presentation of the full model documents how many coefficients were estimated and the proportion of estimates that attained significance. Since every insignificant effect estimate constitutes a theory component lacking evidentiary support from the current data, there is substantial incentive to avoid introducing multiple coefficients that would constitute fishing for significant effects.

Second, fishing might apply to the overall model χ^2^ test. In the context of model fit, the fishing would be reverse-fishing, or fishing-for-insignificance, but selection of randomly produced insignificant-ill-fit results could still introduce testing bias. This seems of little concern because researchers rarely have multiple theories and hence do not develop enough different models to constitute fishing for model insignificance. Dropping insignificant coefficients also biases model testing toward reporting fit (by increasing the χ^2^ degrees of freedom proportionately more than the model’s χ^2^ value) but this practice is often relatively ineffective and seems to be generally on the decline (except in factor analytic contexts).

The third concern is the most cogent, and recommends reconsidering both the prior concerns for fishing. When a model fails, researchers routinely examine the modification indices to see whether introduction of specific model coefficients would improve model fit. A coefficient that significantly improves model fit will, if freed, also result in a statistically significant coefficient estimate. Hence, scanning the modification indices for coefficients capable of significantly improving model fit constitutes a fishing expedition. Selective inclusion of data-prompted coefficients increases concern for both coefficient-fishing and model-fishing.

Fortunately, a variety of common procedural admonitions mitigate these concerns.

- Only coefficients consistent with the modeled theory should be added at the behest of modification indices. A theory that is consistent with just about any additional coefficient is just about no theory at all.
- Only a very limited number of post-hoc coefficients should be added – three seems about the limit [27].
- The full history of model modifications should be reported – which promotes theory-consistent modifications, and makes it possible to assess the seriousness of this concern.
- When scanning the modification indices, only truly large modification indices should be considered, and not those near the 4.0 boundary of statistical insignificance, because considering larger modification indices reduces the risk of capitalizing on purely random variations. No specific value can be provided for the size of modification indices warranting attention but in practice a reasonable value can be obtained by remembering that only about three model modifications can reasonably be made to a model [27]. Hence, two or three model modifications should reduce χ^2^ from its current value to a value somewhere near the middle of the relevant χ^2^ distribution (which equals the model degrees of freedom). Thus, modification indices as large, or larger, than about one-third the difference between the current χ^2^ and the model degrees of freedom are worth considering. Only properly-signed estimates should be introduced, and special caution is warranted for measurement error covariances because the measurement error variables in structural equation models are beyond, or are external to, the substantive modeled latent theory, and hence covariances between error variables often lack substantive theoretical grounding. Modification indices attached to measurement error covariances are prone to “recommending” theoryless reductions in covariance ill fit because measurement errors are disconnected from the latents comprising the modeled theory.

These ameliorative procedures and cautions are available, but if they are not heeded “fishing” remains a concern in structural equation model testing. The more frequent and less-theoretical the modification index induced changes, the greater the concern even if the model subsequently fits. If a model remains significantly ill fitting despite modification-index-induced changes, that constitutes especially strong and worrisome evidence of model problems.

### Argument 6: p = 0.06 versus 0.04

This argument against model testing points to 0.05 as an arbitrary artificial fit/fail boundary that claims nearly identical data-based evidence (*p* = 0.06 versus 0.04) deserves radically different researcher responses (namely accepting versus rejecting the model). The 0.05 boundary is artificial because it corresponds to one point on a smoothly declining sampling distribution. It is arbitrary because a variety of other boundaries might have been chosen – the probabilities 0.10 and 0.01 come to mind. Supporting model testing does not require challenging either the arbitrariness or artificiality of the 0.05 boundary. It requires, in one way challenging and in another way encouraging a radically different researcher response.

When a model significantly fails by having a χ^2^*p* value less than 0.05, the model is displaying an amount of covariance ill fit likely to arise rather infrequently (namely with probability <0.05) via purely random variations. The small *p* value recommends diagnostic investigation of the full range of non-random reasons for ill fit – most importantly reconsideration of the possibility of model causal misspecification, but also rechecking: the model’s programming, the missing value specifications, any data manipulation/recoding, the possibility of other human-errors, and so on. These things should have been checked even if the model had fit, rather than failed. Thus, in a variety of regards a conscientious researcher’s actions slightly to one side of 0.05 should not differ radically from their actions on the other side of the 0.05 boundary. Model tests with *p* values of 0.04 and 0.06 should not prod *conscientious* researchers into radically different research behavior or into making radically different claims about the model. There either is enough, or is almost enough, evidence to speak against the model.

What surpassing the 0.05 boundary does is untether the academic watchdogs and sic them on any researcher who is not so conscientiously inclined. Significant model failure signals that the excuse of “merely random variations” has declined sufficiently that acknowledgment and diagnostic investigation of the other possibilities is required, and demanded by the academic community, not merely recommended or left to the researcher’s discretion. At 0.06, 0.07 and 0.08 the researcher should have been proactively considering, investigating, and acknowledging the full range of possible problems. Surpassing 0.05 merely frees the wider academic community to demand what should have been done voluntarily. Thus the 0.05 boundary does not make any radical difference to the behavior or claims of conscientious proactive SEM researchers. It merely signals when *inattention to the relevant methodological concerns and alternatives* would constitute methodological inadequacy warranting pointed reviewer criticism and reputation tarnish.

### Argument 7: statistical assumptions

The statistical assumptions on which χ^2^ is based have been investigated and found to be basically robust but with some inevitable limitations – limitations which some people employ as excuses for disregarding χ^2^ testing, rather than adopting more appropriate corrective procedures. Chief among these is the presumption of a multivariate-normal indicator distribution. Non-normal distributions can incline χ^2^ to over-reject true models but a compensating adjustment was developed by Satorra and Bentler [12, 28] and is currently available in most SEM programs, so this excuse is unpersuasive if the adjusted χ^2^ is required and used. Similarly, nonlinearities are most effectively addressed by appropriate rescaling of the relevant variables, not by discarding model testing; and discontinuities in indicators’ measurements may be addressed by employing appropriate estimators and testing procedures [29].

An infrequently voiced, but nonetheless real, statistical concern arises because χ^2^ is somewhat sensitive to small *N*. The discussion of Argument 4 noted that χ^2^ behaves appropriately for large *N*, but there remains the concern that χ^2^ tends to slightly over-reject true models when *N* is small (e.g. [23]: Table three). At *N* = 100, the amount of over-rejection is noticeable but quite small, though the precise extent of over-rejection depends on the actual *N* and the form of the model [30]. It would be inappropriate to use such observations to justify routine disregard for χ^2^ testing because this concern applies only to small-*N* contexts, and only to models coming reasonably close to satisfying the traditional 0.05 alpha for the χ^2^ test.

### Argument 8: it is impossible to find real fitting models

The multitude of failing structural equation models – especially factor models – inclined some people to argue that it is impossible to find models that fit real data without engaging in statistically dubious pursuits like adding modification-index prompted measurement error covariances. This argument became prominent on SEMNET in the context of discussing Browne, MacCallum, Kim, Andersen and Glaser [31]. Browne, et al. sought the mathematical foundations for why χ^2^ is sensitive – according to them overly-sensitive – to problems in structural equation models. They illustrated the supposed over-sensitivity of χ^2^ with a model of laboratory data on natural killer cell activity that provided close yet significantly poor covariance fit. Their sophisticated discussion of the statistical foundations of χ^2^’s power to detect problems, and the focus on a complex cancer related research topic, seemed convincing – until the supposed over-sensitivity of the χ^2^ test vanished when Hayduk, Pazderka-Robinson, Cummings, Levers, and Beres [32] cleanly fit the same data using a structural equation model incorporating an additional feature of the laboratory procedure. The χ^2^ test had not been overly-sensitive; it was displaying substantial power to detect correctable problems. Cleanly fitting models have been reported in a variety of substantive areas [32–35], and there have been reports of a single model cleanly fitting several systematically-differing data sets [5]. Enough substantive and cleanly fitting structural equation models have been found to render this supposed-impossibility actually possible. The χ^2^ test is not prefect – it cannot detect misspecified covariance-equivalent models – but it stands as the strongest available model test, and as a test capable of detecting many correctable modeling mistakes.

### Argument 9: there is no assured way to fix failing models

It is true that there is no assured way to fix each and every model that testing detects as problematic. The available SEM diagnostics are simply insufficient to unerringly pinpoint which of the many things that could go wrong, have gone wrong in any given instance. This “argument” amounts to saying there is no diagnostically-guaranteed route to attaining proper scientific understandings. Fortunately, science functions adequately in contexts where the evidence points to current deficiencies, even when that evidence is not accompanied by diagnostics assuredly directing the researcher toward a superior understanding. Researchers unwilling to respect evidence of deficiencies in their current understandings are unlikely to dedicate themselves to overcoming those deficiencies. There being no clear and assured roadmap to scientific advancement via SEM does not justify disregarding evidence of SE model problems.

### Argument 10: editorial bias and researcher dishonesty

The observation that journal editors are disinclined to publish failing models is translated by some people as implicit instruction to not report their model as failing. It seems reasonable, even laudable, for editors to reject ridiculous ill-conceived models that fail, because rubbish is not worth publishing. But it is an entirely different matter to refuse to publish theoretically based and conscientiously constructed structural equation models that fail. Routinely refusing to publish substantive failing models can create disciplinary dead-ends because the discipline is not being informed of substantive theoretical and/or methodological deficiencies. Hayduk, Cummings, Boadu, Pazderka-Robinson and Boulianne [9] argue “that attentively constructed and theoretically meaningful models that fail ought to be carefully discussed and published” because science will move in new theoretical and methodological directions when the current theories and methods are demonstrably inadequate. The difficult challenge is to separate the theoretically based and substantively informative yet failing models from models that failed because they were ill conceived nonsense. Here journal editors are dependent on reviewers competent in both the relevant substantive theory and SEM’s statistical methodology. Fortunately, SEM has matured to the point where there are such people in a variety of substantive fields.

The obverse of this issue rests on the author’s shoulders. How should an author respond to a failing structural equation model? The author with a truly substantive model should not hesitate to report both the model’s failure and their successful/unsuccessful diagnostic investigations of the various potential reasons for failure. It is authors having failing theory-weak models whose academic honesty will be plumbed by the temptation to “forget” to report the model’s failure, or to “excuse” the failure by pointing to some close-fit index, or to uncritically follow modification indices until the model fits. These temptations are likely to be especially intense if the researcher’s own theory is confronted by the evidence.

Anyone thinking the challenge to honestly report uncomfortable test results is a trivial or infrequent concern should remember that fit indices were initially introduced precisely to mollify researchers fretting over failing models. In the festschrift for Karl Joreskog, Dag Sorbom provided the following anecdote regarding the χ^2^ test.

“*Another one [anecdote] is from about 1985 when we gave a LISREL workshop. At that time, when use of the new methodology was not widespread at all, there were many LISREL applications producing large or huge chi-squares. We had just added GFI and AGFI to the program. In his lecture Karl would say that the chi-square is really all you need. One participant then asked “Why have you then added GFI?” Whereupon Karl answered “Well, users threaten us saying they would stop using LISREL if it always produces such large chi-squares. So we had to invent something to make people happy. GFI serves that purpose.””*

([6]:10, *[ ]* added).

Researcher threats and biases are not really “arguments,” and it should be clear that appeals to fit indices can constitute shameful disrespect for χ^2^ test evidence of model problems. Editors would serve their readership well if they strived for scrupulously-honest assessments of the structural equation models appearing in their journals.

## Summary and discussion

The melding of factor and path analysis under structural equation modeling proceeded statistically smoothly but factor-analytic-based procedures that implicitly disregarded model testing, aided by the Wilkinson, et al. [2] report, spread a lamentable disregard for evidence of model failure from psychology and its companion disciplines into SEM more generally. The “theoretical elasticity” of factors being retrospectively labeled as features common to item sets, rather than being specific theorized latent causes of items [36, 37], inclined factor models to be seen as nil-null hypotheses, rather than theorized and notable-null hypotheses. This theory laxity, supplemented by entrenched but weak factor rules of thumb, resulted in so many failing models that factor-based researchers readily adopted a host of test-displacing fit indices rather than address significant model failures. The disregard of SEM test evidence lacked justification, though the flawed arguments discussed above camouflaged the disregard of troubling evidence for decades and permitted the evidence-disrespect to spread surprisingly widely. The consequences of disrespecting evidence of structural equation model failure has contaminated many areas but will be especially difficult to expunge from disciplines in which problematic factor models supposedly “supported” the construction of measurement scales. The disregard of model-challenging evidence has left areas historically dependent on factor models confronting mountains of untrustworthy literature and mired in a complicated academic mess. How is a researcher to proceed if half the items in a scale do not reflect the latent factor supposedly being measured? Clearly each afflicted area will have to find its own way out of its measurement conundrums.

To keep these comments from appearing as personal attack, we have not referenced specific evidence-shy researchers – but we are willing to provide 20 relevant citations, at the editor’s or reviewers’ request. Neither have we listed all the indices used to detract from structural equation model testing. It should be clear from the discussion preceding Argument 2 above that the amount of covariance ill fit, by *any* covariance-based index, cannot be trusted to correspond to the seriousness of a model’s misspecifications because it is possible for even seriously causally misspecified models to provide perfect covariance fit. Statisticians, SEM programmers, and SEM-text authors should be apologetic for not warning of the deficiencies of fit indices as checks on model misspecification [15]. Statisticians and programmers can legitimately claim they are not responsible for policing inappropriate use of whatever indices they create or present, but they must nonetheless shoulder responsibility for failing to provide clear warnings that indexed amounts of covariance ill fit cannot be trusted to correspond to the seriousness of model misspecification. Indices have legitimate uses, but they are inappropriately transformed into tests when specific index values are proposed as signifying “acceptable fit” – which becomes “acceptable model fit” and progresses to the “model is acceptable according to the index”. The reasonable intentions of some index developers have been sullied by those condoning, rather than confronting, the indefensible interpretational slide from indices reporting “covariance fit” to reporting “the acceptability of the model”. The lion’s share of responsibility, however, rests on researchers inappropriately using fit-index values to distract from the evidence of model failure. This article should remind researchers of their responsibility.

Researchers confronting the SEM-testing quagmire can expect to confront a variety of difficulties as they attempt to return to honest and appropriate structural equation model testing. Senior researchers are likely to encounter difficulty polishing their reputations while acknowledging and correcting a personal history of disrespect for test evidence. Junior researchers will undoubtedly find it awkward to challenge an entrenched literature. Some budding researchers may even have to “reassess” their own Ph.D. supervisor’s work – at the risk of personality conflict, a thesis battle rather than thesis defense, and traitor-worthy letters of recommendation.

Cleaning-up the model-based theories and reassessing factor-based scales is likely to take decades, as retirements, manuscript-rejections, and public shaming, reintroduce respect for SEM test evidence. Two fundamental points are sufficient to propel and sustain the decontamination. First, the tested null hypothesis is not a nil hypothesis – it is as notable, substantive, and worthy as is the theory grounding the model. Thus the test result addresses substantive issues and may challenge substantive claims. And second, the amount of covariance ill fit of a model, as recorded by *ANY* index does not trustably correspond to the seriousness of the problems in a model. Beyond-chance test evidence of inconsistencies between the structural equation model and the data demands diagnostic investigation of what might have gone wrong: in the data, in the modeling process, and/or in the theory itself. Data problems include: forgetting to specify missing values, the use of incorrect but similarly-named variables, omitted or unnecessary rescaling of variables, and incorrect selection of subsets of cases. Modeling-process problems include: mistakes in specifying the model syntax, inattention to program defaults, overlooking failure of the statistical estimator to converge (e.g. when model coefficients are underidentified), use of inappropriate estimators (e.g. for ordinal variables), impossible estimates (like negative variances), and inappropriately fixed values. Theory problems can arise from: incorrect latents (e.g. when additional latents are required), incorrect connections between latents and their indicators, incorrect connections among the various latent variables, and failing to model group-level causal differences (e.g. between males and females). If the χ^2^ test reports failure, several of these things may have gone wrong, so diagnostic investigation of the various alternatives can be quite demanding. The SEM investigator should: pay attention to the methodology that provided their indicators, set up their model carefully, understand their statistical estimator, and pay special attention to the full range of reasonable model alternatives [14, 35, 36].

The χ^2^ test is not perfect. It cannot always detect all the problems in a model – it cannot detect causally-wrong fit-equivalent models, and may only weakly detect other problems. But χ^2^ is the strongest available test and hence provides the most trustworthy evidence of model misspecification or other problems. Disrespect of evidence of problems is sufficient to result in manuscript-rejection and public shame, so we urge everyone: to build their causal structural equation models carefully, to respect the evidence provided by the strongest available test (currently the χ^2^ test), and to report honestly.

### Ethics

No ethics review was required because no human or animal subjects were involved.

## References

[1] Rodgers JL (2010). The epistemology of mathematical and statistical modeling: A quiet methodological revolution. Am Psychol.

[2] Wilkinson L, The Task Force on Statistical Inference (1999). Statistical Methods in Psychology Journals: Guidelines and Explanations. Am Psychol.

[3] SEMNET: **The Structural Equation Modeling Discussion Network.** http://www.aime.ua.edu/cgi-bin/wa?A0=SEMNET

[4] Joreskog K, Sorbom D (1976). LISREL III: Estimation of Linear Structural Equation Systems by Maximum Likelihood Methods, User’s Guide.

[5] Entwisle DR, Hayduk LA, Reilly TW (1982). Early Schooling: Cognitive and Affective Outcomes.

[6] Sorbom D, Cudeck R, du Toit S, Sorbom D (2001). Karl Joreskog and LISREL: A personal story. Structural Equation Modeling: Present and Future. A Festschrift in Honor of Karl Joreskog.

[7] Hayduk LA, Glaser DN (2000). Jiving the four-step, waltzing around factor analysis, and other serious fun. Struct Equ Model.

[8] Barrett P (2007). Structural equation modelling: Adjudging model fit. Person Individ Differ.

[9] Hayduk LA, Cummings GG, Boadu K, Pazderka-Robinson H, Boulianne S (2007). Testing! Testing! One, two, three – Testing the theory in structural equation models!. Person Individ Differ.

[10] Bollen KA (1989). Structural Equations with Latent Variables.

[11] Hayduk LA (1987). Structural Equation Modeling with LISREL: Essentials and Advances.

[12] Satorra A, Bentler PM (2010). Ensuring positiveness of the scaled difference chi-square test statistic. Psychometrika.

[13] Ledermann W (1937). On the rank of the reduced correlation matrix in multiple-factor analysis. Psychometrica.

[14] Hayduk LA (2014). Seeing perfectly fitting factor models that are causally misspecified: Understanding that close-fitting models can be worse. Educ Psychol Meas.

[15] Savalei V (2012). The relationship between root mean square error of approximation and model misspecification in confirmatory factor analysis models. Educ Psychol Meas.

[16] Heene M, Hilbert S, Freudenthaler HH, Buhner M (2012). Sensitivity of SEM fit indexes with respect to violations of uncorrelated errors. Struct Equ Model.

[17] Browne MW, Cudeck R (1992). Alternative ways of assessing model fit. Soc Method Res.

[18] Hu L, Bentler PM (1999). Cutoff criteria for fit indexes in covariance structure analysis: Conventional criteria versus new alternatives. Struct Equ Model.

[19] Box GEP, Launer RL, Wilkinson GN (1979). Robustness in the strategy of scientific model building. Robustness in Statistics.

[20] Box GEP, Draper NR (1987). Empirical Model-Building and Response Surfaces.

[21] Box GEP (1979). Some problems of statistics and everyday life. J Amer Stat Assoc.

[22] Joreskog K, Sorbom D (1996). LISREL 8 User’s Reference Guide.

[23] Fan X, Thompson B, Wang L (1999). Effects of sample size, estimation methods, and model specification on structural equation modeling fit indexes. Struct Equ Model.

[24] Box GEP, Hill WJ (1967). Discriminating among mechanistic models. Technometrics.

[25] Box GEP (1976). Science and statistics. J Amer Stat Assoc.

[26] Benjamini Y, Hochberg Y (1995). Controlling the false discovery rate: A practical and powerful approach to multiple testing. J Royal Stat Soc.

[27] Herting JR, Costner HL (2000). Another perspective on “the proper number of factors” and the appropriate number of steps. Struct Equ Model.

[28] Satorra A, Bentler PM, von Eye A, Clogg C (1994). Corrections to test statistics and standard errors in covariance structure analysis. Latent Variable Analysis in Developmental Research.

[29] Muthen BO, Bollen KA, Long JS (1993). Goodness of fit with categorical and other nonnormal variables. Testing Structural Equation Models.

[30] Curran PJ, Bollen KA, Paxton P, Kirby J, Chen F (2002). The noncentral chi-square distribution in misspecified structural equation models: Finite sample results from a Monte Carlo simulation. Multivar Behav Res.

[31] Browne MW, MacCallum RC, Kim CT, Andersen BL, Glaser R (2002). When fit indices and residuals are incompatible. Psychol Meth.

[32] Hayduk LA, Pazderka-Robinson H, Cummings GG, Levers MD, Beres MA: **Structural equation model testing and the quality of natural killer cell activity measurements.** *BMC Med Res Methodol* 2005.,**5**(1)**:** doi:10.1186/1471–2288–5-1 10.1186/1471-2288-5-1 PMC546216 15636638

[33] Hayduk LA (1994). Personal Space: Understanding the Simplex Model. J Nonverbal Behav.

[34] Hayduk LA, Stratkotter RF, Rovers MW (1997). Sexual orientation and the willingness of Catholic seminary students to conform to church teachings. J Sci Stud Relig.

[35] Hayduk LA, Pazderka-Robinson H, Cummings GG, Boadu K, Verbeek EL, Perks TA (2007). The weird world, and equally weird measurement models: Reactive indicators and the validity revolution. Struct Equ Model.

[36] Hayduk LA, Littvay L (2012). Should researchers use single indicators, best indicators, or multiple indicators in structural equation models. BMC Med Res Methodol.

[37] Hayduk LA, Pazderka-Robinson H, Outhwaite W, Turner S (2007). Fighting to understand the world causally: Three battles connected to the causal implications of structural equation models. Sage Handbook of Social Science Methodology.

[CR38] The pre-publication history for this paper can be accessed here: http://www.biomedcentral.com/1471-2288/14/124/prepub

